# Characteristics and Impact of Drug Detailing for Gabapentin

**DOI:** 10.1371/journal.pmed.0040134

**Published:** 2007-04-24

**Authors:** Michael A Steinman, G. Michael Harper, Mary-Margaret Chren, C. Seth Landefeld, Lisa A Bero

**Affiliations:** 1 San Francisco Veterans Affairs Medical Center, San Francisco, California, United States of America; 2 Division of Geriatrics, University of California, San Francisco, California, United States of America; 3 Department of Dermatology, University of California, San Francisco, California, United States of America; 4 Department of Clinical Pharmacy, University of California, San Francisco, California, United States of America; 5 Institute for Health Policy Studies, University of California, San Francisco, California, United States of America; Harvard University, United States of America

## Abstract

**Background:**

Sales visits by pharmaceutical representatives (“drug detailing”) are common, but little is known about the content of these visits or about the impact of visit characteristics on prescribing behavior. In this study, we evaluated the content and impact of detail visits for gabapentin by analyzing market research forms completed by physicians after receiving a detail visit for this drug.

**Methods and Findings:**

Market research forms that describe detail visits for gabapentin became available through litigation that alleged that gabapentin was promoted for “off-label” uses. Forms were available for 97 physicians reporting on 116 detail visits between 1995 and 1999. Three-quarters of recorded visits (91/116) occurred in 1996. Two-thirds of visits (72/107) were 5 minutes or less in duration, 65% (73/113) were rated of high informational value, and 39% (42/107) were accompanied by the delivery or promise of samples. During the period of this study, gabapentin was approved by the US Food and Drug Administration only for the adjunctive treatment of partial seizures, but in 38% of visits (44/115) the “main message” of the visit involved at least one off-label use. After receiving the detail visit, 46% (50/108) of physicians reported the intention to increase their prescribing or recommending of gabapentin in the future. In multivariable analysis, intent to increase future use or recommendation of gabapentin was associated with receiving the detail in a small group (versus one-on-one) setting and with low or absent baseline use of the drug, but not with other factors such as visit duration, discussion of “on-label” versus “off-label” content, and the perceived informational value of the presentation.

**Conclusions:**

Detail visits for gabapentin were of high perceived informational value and often involved messages about unapproved uses. Despite their short duration, detail visits were frequently followed by physician intentions to increase their future recommending or prescribing of the drug.

## Abstract 2

Visits from pharmaceutical representatives regarding gabapentin "detailing" were frequently followed by physician intentions to increase their future activity with the drug.

## Editors' Summary

**Background.:**

In the US, before a pharmaceutical company can market a drug to doctors for use in a specific “indication” (meaning the treatment for a particular disease and group of patients), the drug has to be approved as safe and effective for that use by a government agency, the Food and Drug Administration. Once approved, doctors are allowed to use a drug for whatever nonapproved indications they think are appropriate, but the drug company cannot actively promote the drug for anything other than its approved use. However, many people are concerned that drug companies indirectly try to promote use of drugs for indications that are not approved. Such illegal activity would help a drug company increase its market share and sell more drugs. One tactic that drug companies use to sell drugs is “detailing.” Detailing involves direct visits from drug company representatives to individual doctors, during which the representative would provide information about their company's drugs. However, not a great deal is known about detail visits and the effect that they have on doctors' attitudes towards the drugs that are being promoted.

**Why Was This Study Done?:**

The researchers carrying out this study wanted to learn more about what happens during detail visits and what impact these visits have on prescribing behavior. An opportunity for researching this came about as a result of a lawsuit during which drug company documents were subpoenaed (i.e., required by the court to be made available). In that lawsuit, it was alleged that a drug company, Parke-Davis, had promoted a drug, gabapentin, for many nonapproved uses. The company that subsequently took over Parke-Davis eventually made an out-of-court settlement. During the relevant time period, the only approved use of gabapentin was for treatment of partial seizures in adults with epilepsy, in combination with other drugs. However, gabapentin was used for many other conditions such as treatment of psychiatric disorders and management of pain. These researchers therefore used the documents available as a result of the lawsuit to research detailing and what impact detailing had on doctors' attitudes towards the drug being promoted.

**What Did the Researchers Do and Find?:**

The documents analyzed in this study were produced by Verispan, a market research company. Verispan asked doctors who had been visited by Parke-Davis sales representatives to fill out a standard form after each detail visit. These forms were then subpoenaed as part of the lawsuit against Parke-Davis. The researchers here focused specifically on data relating to visits made by a single sales representative to a doctor or small group of doctors, and collected 116 forms. The data available from these forms included the doctors' ratings and comments regarding the main message associated with the products; the informational value of the visit; the quality of the presentation; and whether the doctor currently prescribed or planned to prescribe the product. The researchers classified the information available from the forms, identifying whether the “main message” related to approved uses of the drug or not; and extracting data relating to whether doctors planned to increase, maintain, or decrease their use of the drug. The majority of the visits studied were to doctors who were not neurologists, and would therefore be unlikely to prescribe gabapentin for its approved use. Doctors reported that a substantial proportion of the detail visits contained messages relating to nonapproved uses of gabapentin. Nearly half the doctors stated in the forms that their use of gabapentin would increase in the future, and no doctors said that their use would decrease following the visit. Doctors' intention to increase their use of gabapentin in the future was similar regardless of whether the message of the visit involved an approved or unapproved use.

**What Do These Findings Mean?:**

This study shows that in the case of gabapentin, detail visits by drug company representatives frequently promoted nonapproved uses of the drug; these visits often resulted in doctors planning to increase their use of gabapentin. However, it is not clear whether these findings are also true for other drugs and drug companies, in part because these data came about as a result of a unique opportunity granted by the lawsuit against Parke-Davis.

**Additional Information.:**

Please access these Web sites via the online version of this summary at http://dx.doi.org/10.1371/journal.pmed.0040134.

- Medline Plus (provided by the US National Library of Medicine) has an entry about gabapentin
- Introductory information is available from the US FDA Center for Drug Evaluation and Research about the drug approvals process in the USA
- Wikipedia has an entry on pharmaceutical marketing (Note that Wikipedia is an internet encyclopedia anyone can edit)
- The Drug Industry Document Archive is available at University of California, San Francisco; this internet archive holds documents relating to the lawsuit against Parke-Davis and from which the data presented in this paper derives
- Guidance is available from the International Federation of Pharmaceutical Manufacturers and Associations regarding ethical promotion of medicines

## Introduction

Visits to physician offices by pharmaceutical sales representatives are among the most visible and effective forms of drug industry promotion [1]. As such, their role has generated substantial debate in the medical community [2–4]. Some commentators have argued that these “details” provide unbalanced information and thus negatively impact prescribing quality, a problem compounded by the influence of gifts that commonly accompany these interactions [5–10]. In addition, negative public perceptions of these relationships may harm the standing of the medical profession. Others have argued that sales representatives provide important information about drugs to physicians, and thus may improve prescribing quality [11–13].

While a number of studies have investigated the frequency of detail contacts and the association between these contacts and prescribing behavior [1,14–21], relatively little has been published in the medical literature on what actually happens during detail visits [22,23]. Studies using physician self-reports suggest a range of attitudes about the educational content of these interactions and the perceived effect of detail visits on prescribing [1,12,15,24–28]. However, these studies may be subject to recall bias, due to reliance on self-reports long after the conclusion of detail visits and a focus on respondents' general impressions rather than on specific interactions. In addition, nearly all data on this topic published in the medical literature have originated from academic settings. These settings may subtly bias survey responses, since physicians may believe industry contacts are judged negatively in the academic community and thus may tailor their answers to be more socially acceptable.

To learn more about the content and potential impact of detail visits, we used data collected from physicians by a market research company about detail visits for gabapentin (trade name Neurontin). These data were obtained from documents subpoenaed in a qui tam whistleblower lawsuit alleging that drug manufacturer Parke-Davis violated federal regulations in the mid- to late-1990s by promoting gabapentin for uses not approved by the US Food and Drug Administration [29]. Over this period, the only approved use of gabapentin was for the adjunctive treatment of partial seizures in persons over 12 years of age at doses up to 1,800 milligrams per day. However, interest in the drug for the management of pain syndromes, psychiatric diseases, and other conditions presented an opportunity for a much wider market, and Parke-Davis used a variety of coordinated strategies to promote gabapentin for both on- and off-label uses [30]. In 2004, this litigation concluded with a US$430 million out-of-court settlement by Warner-Lambert, Parke-Davis' parent corporation [29].

Using data from documents subpoenaed in this litigation, we describe the characteristics and content of detail visits for gabapentin and the self-reported impact of these details on future prescribing of and attitudes toward the drug. Next, we examine which aspects of details were associated with physicians' intention to increase their future prescribing or recommendation of gabapentin.

## Methods

### Data Source and Inclusion Criteria

All data used in this study were subpoenaed from Verispan, a health care information company whose specialties include market research for pharmaceutical companies, for use in United States of America ex rel. David Franklin v. Pfizer, Inc, and Parke-Davis, Division of Warner-Lambert Company [31]. Data on drug detail visits were collected by Verispan, which recruited physicians in certain specialties throughout the United States to serve as “panelists” [32]. At the conclusion of each detail visit by a pharmaceutical sales representative, panelists were asked to complete a brief standardized form (known as a “verbatim” or “detailing reporting form”) that described characteristics of the visit. Physicians were compensated by Verispan for their participation in this program, but had no other contact with the company, and were given guarantees of confidentiality [32]. Data collected in this way are typically purchased by pharmaceutical firms for market research purposes [32].

Forms produced by Verispan in response to subpoena (which requested all data pertaining to gabapentin from 1994 through 2002) were obtained from the US District Court in Massachusetts and from the law firm that represented the whistleblower plaintiff, and form the basis for this report. In this study, we sought to focus on the “classic” type of pharmaceutical detail, i.e., a visit from a sales representative to a single physician or a small group of physicians. In the absence of a commonly accepted definition, we defined a classic detail as a visit by an industry sales representative to an individual physician or group of up to three physicians in the workplace setting. Among 142 forms, we excluded 26 that appeared to describe other types of encounters, including 16 visits in which more than three physicians participated, four visits in which more than one company was involved, three visits that occurred in a nontraditional location or format (“inservice/[continuing education] program”), and three visits whose main message suggested that the encounter occurred via teleconference. The remaining 116 forms composed our analytic sample, and included three variations of the same data collection instrument.

The 116 forms were contributed by 97 physicians, including 87 physicians who contributed one form, six physicians who contributed two forms each, and four physicians who completed three to seven forms each (with each form representing a separate visit). As our primary interest was in the detail visit itself rather than the individual physician, our primary analyses evaluated all 116 forms. Among the 116 forms, 91 (78%) were from 1996; eight (7%) were from other years (1995 and 1997–1999), and the remaining 17 (15%) forms did not provide complete date information. It is unclear why the majority of forms provided were from a single year, although the absence of other years may be related to company practice of destroying forms more than several years old [32]. A spreadsheet provided in response to the subpoena shows that an additional several hundred forms were completed for encounters between 1995 and 1998, but these data were not usable due to the limited amount of information provided and the uncertain context of these interactions [31].

**Figure 1:**
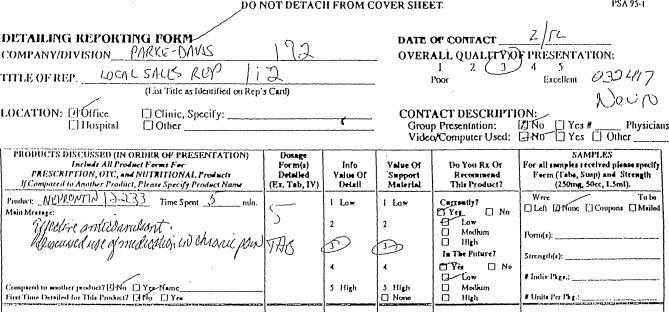
Detailing Reporting Form Excerpt from a detailing reporting form providing information on a detail visit for gabapentin. Some markings (e.g., “032417 Neuro” and “12233” next to “Product: Neurontin”) were annotations entered by Verispan employees to assist with coding and data entry of completed forms.

Information provided by the physician on verbatim forms (Figure 1) included the date and location of the visit, whether it was a one-on-one or group visit, the main message associated with each product (entered as free text), and the informational value of the detail for each product as well as the overall quality of presentation for the entire visit (recorded on a five-point Likert scale with anchors of “poor” and “excellent”). In general, physicians did not provide any other comments in the free text space. Physicians were also asked whether they prescribed or recommended the product and at what level (“low,” “medium,” or “high”), with separate responses for current and expected future practice. (On six forms, similar information was collected using a different set of questions). Physicians were asked to send in their reports monthly, and an estimated 90% of reports were filed within one to two months after the visit [32].

### Data Extraction

Using free text entered in the “Main Message” field of the market research form (Figure 1), two of the authors (MAS, GMH) independently classified the main message for each detail visit into one or more clinical indications (see Box 1). Disagreements were resolved by consensus, with additional adjudication by the senior author (LAB) when necessary. For comparative analyses, visits were categorized into three mutually exclusive categories based on the type of messages communicated. These included (1) messages that mentioned the approved indication; (2) messages that mentioned uses not approved by the FDA; and (3) messages that mentioned a mix of approved and unapproved uses.

While physicians are allowed to prescribe medications for any purpose they see fit, drug manufacturers are allowed to promote drug use only for indications that have been FDA approved [29]. Thus, we considered any mention of seizure or epilepsy to be an approved message, except messages which specifically mentioned gabapentin as monotherapy for epilepsy, which was unapproved. Messages containing other content (e.g., comments about pharmacokinetics, drug–drug interactions, or nonspecific comments) could also be present in any of the three categories. However, if the only message(s) present was this other content, we classified the visit message as approved. Of note, in 2002 gabapentin received FDA approval as treatment for post-herpetic neuralgia, but because the study period was well before this, we considered this use to be “off-label” in our analyses.

To evaluate which characteristics of the detail visit were associated with the intent to increase prescribing or recommendation of gabapentin in the future, we constructed a change measure for each physician. Physicians who intended to change from nonusers to users or who intended to increase their current level of use were classified as intending to increase their future use. Corresponding logic was used to identify physicians who intended to maintain or decrease their future use. As no physicians reported an intended decrease in their future use of gabapentin, the change measure was dichotomized into increased versus stable use. Seventeen physicians reported “high” levels of gabapentin prescribing or recommending at baseline and were excluded from some of our analyses because there was no reliable way to detect a future increase (i.e., a “ceiling effect”). All 17 of these physicians reported the intention to continue prescribing or recommending the drug at “high” levels in the future.

### Statistical Analyses

We conducted bivariate analyses using Chi-square and Fisher exact tests to evaluate characteristics associated with an intention to increase future prescribing or recommending of gabapentin. We then constructed a multivariable model using backward stepwise selection of these variables, with *p* < 0.20 to stay in the model. After stepwise selection, we used the covariance matrix of the parameter estimates to identify and remove highly collinear variables. To preserve power in this analysis, missing data were coded as a separate category for each variable. These categories of missing data are not reported. On sensitivity analysis of the final model, results were not substantively different when these “missing” categories were reclassified as each of the possible levels of the corresponding variable, or recoded as missing data with loss of the observation.

We conducted two additional sensitivity analyses. First, to restrict our sample more specifically to one-on-one encounters, we repeated our analyses after excluding 11 visits in which two or three physicians participated, and 25 visits in which data were not provided on the individual versus group nature of the encounter. Second, we repeated our analyses after excluding multiple visits to the same physician (each recorded on a separate form), keeping only the form with the earliest date for a given physician.

Analyses were conducted using Stata 8.2 (http://www.stata.com). This research was approved by Research and Development Committee of the San Francisco Veterans Affairs Medical Center and the Committee on Human Research of the University of California, San Francisco.

## Results

### Characteristics of Details and Events

Characteristics of the 116 visits in our analytic sample are shown in Table 1. Over half of detail visits (56%) were to physicians in fields other than neurology, the majority of which (46/63) comprised visits to doctors of internal medicine, family or general practice, or osteopathic medicine. The median visit duration was 5 min. Among a subset of 33 forms in which physicians were asked about formulary status for gabapentin, 30 physicians (91%) responded that the drug was on their hospital's formulary and the remaining three (9%) did not know.

**Table 1:**
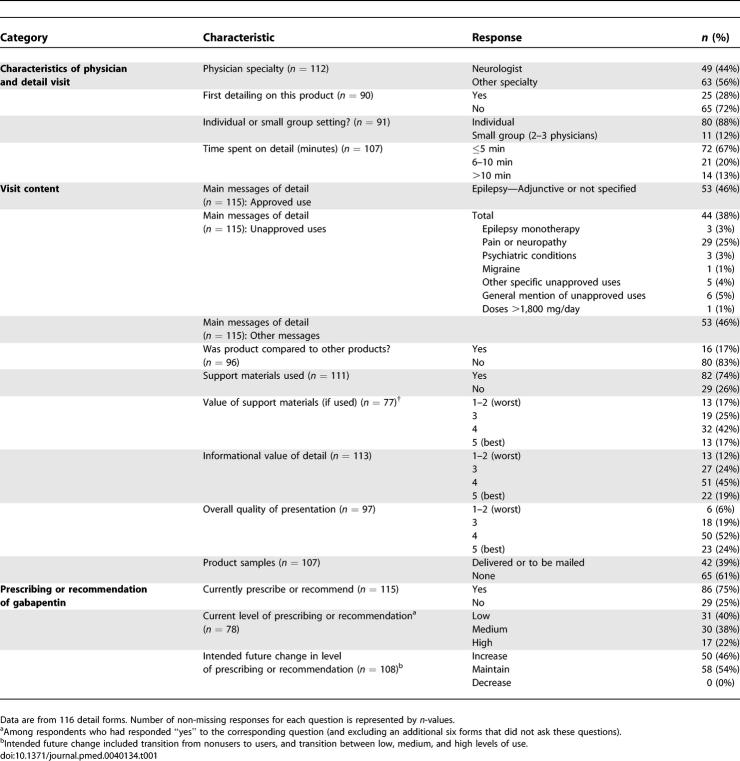
Characteristics of 116 Detail Visits

Physicians recorded in their own words a number of distinct “main messages” associated with the detail visit for gabapentin. Unapproved messages were present in 44 (38%) of visits, including 26 visits (23%) whose “main message” cited only unapproved uses, and 18 visits (16%) that mentioned both approved and unapproved uses. Neurologists and non-neurologists reported a similar proportion of approved messages, unapproved messages, and messages containing both approved and unapproved content (Chi-square statistic 2.02, *p* = 0.36 for difference in message type between specialties).

Physicians rated the informational value and overall quality of the detail visits highly (Table 1). There was a nonsignificantly lower perceived informational value for detail visits that focused on unapproved messages. On a five-point Likert scale (with 5 being best), physicians rated the informational value as 4 or 5 in 46% (12/26) of details with only unapproved messages, 71% (12/17) of details with a mixture of approved and unapproved messages, and 70% (49/70) of details with approved messages (Chi-square statistic 5.03, *p* = 0.08 for difference in value rating between message types).

### Factors Associated with Intention to Increase Future Prescriptions or Recommendations for Gabapentin

Overall, 46% (50/108) of physicians stated that their prescribing or recommending of gabapentin would increase in the future. No physicians reported the intention to decrease their future use or recommendation of the drug. On bivariate analyses, physicians' intention to increase prescribing or recommending of gabapentin was higher among non-neurologists, physicians with a lower current level of prescribing, and for visits that occurred in small group (versus one-on-one) settings (Table 2). There was also a nonsignificantly higher intention to prescribe among physicians who received visits that lasted longer than 5 min (*p* = 0.08) and among physicians who perceived the visit to be of high informational value (*p* = 0.07). On multivariable analysis, receiving the detail visit in a small group setting and low baseline use or recommendation of gabapentin were the only variables associated with the intention to increase future activity with the drug (Table 2).

**Table 2:**
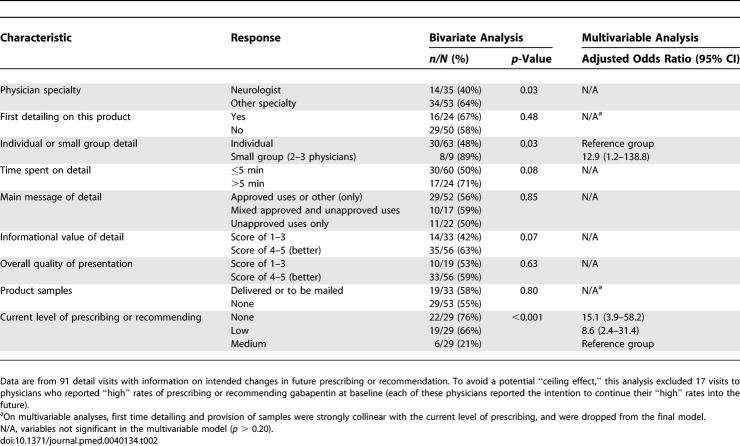
Factors Associated with Intention to Increase Future Prescribing or Recommending of Gabapentin

### Sensitivity Analyses

When the sample was restricted to the 80 physicians with known one-on-one details, there were no substantive differences in our results. Similarly, when we restricted the sample to include only one form per physician (*n* = 97), our analyses were not substantively changed.

## Discussion

In this study of drug detail visits for gabapentin, we found that most detail visits were brief and of high perceived quality, and often discussed unapproved uses of this drug. Moreover, after receiving the detail visit, half of participants reported the intention to increase their future prescribing or recommendation of gabapentin.

The high perceived quality of these presentations is consistent with the findings of many (but not all) previous studies of physician attitudes toward pharmaceutical representatives [1,25,33–40]. However, the perceived quality of these presentations may not reflect the validity of their educational content. For example, some of the off-label uses for which gabapentin was promoted are not well supported by high-quality clinical trial evidence [41]. Other uses such as neuropathic pain and migraine prophylaxis have been found to be effective in controlled clinical trials, yet these trials had not been published during the period that we studied (aside from two trials published in the final month of 1998, near the end of our study period) [42–44]. More generally, studies suggest that incorrect or misleading information about drugs may frequently be conveyed in promotional settings [5–7,10,23,45,46], that physicians do not consistently distinguish between correct and incorrect information [5,7,47,48], and that it is the perceived (rather than actual) quality of information that produces behavior change [49,50]. In our study, the association between perceived informational value of details and self-reported behavior change was in the expected direction, but did not meet statistical significance.

In addition to the credibility of the information provided at detail visits, the interpersonal aspect of interactions between pharmaceutical representatives and physicians has been hypothesized to be a major driver of behavioral change, and has been emulated with good success by “academic detailing” [1,8–10,22,51–54]. Unfortunately, the forms used for this analysis provide little information about this interpersonal content and its relationship to future behavioral change. Among the visit characteristics that we were able to measure, physicians who received details in small group settings were more likely to increase their future use or recommendation of gabapentin than physicians receiving one-on-one details. This may reflect a “group-think” mentality whereby the perception that one's colleagues are receptive to the detail visit increases one's own receptivity [22,55–57].

The variables that were not associated with future behavior change may be as interesting as those that were. In particular, physicians reported similar increases in future prescribing or recommending of gabapentin after exposure to approved or unapproved messages, suggesting potential susceptibility to discussion of new and relatively unproven uses. Also, visit duration was not associated with the frequency of future behavior change on bivariate analyses. While the failure to detect an association may be due to our limited sample size, even so 50% of these brief visits were associated with reports of increased future use or recommendation of gabapentin. This suggests that even brief encounters may have substantial impact. This may reflect the success of sophisticated marketing techniques that enable pharmaceutical representatives to track the prescribing patterns of individual physicians and to succinctly deliver tailored marketing messages, employ influence techniques, and regulate the content of the visit all the way down to the firmness and duration of the introductory handshake [10,22,58,59].

While we are unaware of other studies published in the medical literature that use methods similar to ours, other reports have found a range of real or perceived impacts of pharmaceutical detailing on prescribing [1]. Several studies have found positive associations between the frequency of interactions with sales representatives and prescribing behavior, and in physician self-report studies approximately one-third to two-thirds of respondents perceive themselves to be influenced by pharmaceutical sales representatives, although more believe that “other physicians” are influenced [15,16,60–62]. Studies using objective data on prescriptions have found a consistently positive impact of detailing on prescribing, often with a greater impact than other forms of marketing such as direct-to-consumer advertisements [1,20]. For example, major effects were observed in a small study of a resident-run psychiatry clinic [19]. Other studies have observed more modest effects, with substantial variation in the effectiveness of detailing for different drugs [18]. The strong effects observed in our study may in part reflect the market position of gabapentin, which at the time of the study was relatively new and of substantial interest for a growing number of conditions, a situation in which the effectiveness of detailing may be greatest [1].

Certain characteristics of detailing for gabapentin may not be universally applicable to detailing for other drugs. Most notably, the promotional campaign for gabapentin involved multifaceted efforts to encourage prescribing for uses not approved by the FDA [29]. As a result, detailing for this drug may have involved an atypically high number of unapproved messages (although a study from France suggests that discussion of unapproved uses may be common for other drugs as well) [23]. In addition, excitement in the medical community about novel uses of gabapentin may have made physicians particularly receptive to these messages.

Other visit characteristics, such as the duration and perceived informational value of the visit, may be more applicable to other drugs. However, our data do not allow us to test this hypothesis, and our results should thus be interpreted as a case study of detailing for one drug. Nonetheless, the focused nature of our data also represents a unique strength, as attention to a single product and individual visits helps avoid the generalizations and recall biases that may affect most existing surveys of pharmaceutical detailing.

Our study has several limitations. First, the perceived acceptability of providing certain answers to the market research firm could have affected physician responses to this self-reported survey or affected their conduct in interactions with pharmaceutical sales representatives. Second, we have limited information about the content of detailing interactions other than a brief “main message” recorded by the physician. Discussion of unapproved uses may have been initiated by the physician. Third, the self-reported intention to increase future prescribing or recommending of gabapentin might have been affected by factors other than the detail. Thus, we cannot prove a causal relationship between the detail and self-reported behavior change, and we do not know whether future intentions became future actions. Fourth, in our multivariable model the presence of prior visits and the distribution of samples were both collinear with the current level of prescribing, which impacted our model construction. Thus, we cannot reliably disentangle the effects of each of these factors on intentions to increase future prescribing. In addition, the small sample size limits the precision of the observed point estimates and our ability to detect all but the strongest associations. Fifth, although we did our best to focus on the classic form of detailing, it is possible that our sample inadvertently included contact reports from other types of interactions. Finally, as described earlier it is unclear why most of the forms provided in request to the subpoena were from 1996, when the period covered under the subpoena was much broader.

**Box 1: Classification of Main Message Content**

Approved indications*

• Seizures, either specified as adjunctive therapy or not specified

**Nonapproved indications or doses**

• Monotherapy for seizures

• Pain or neuropathic syndromes

• Psychiatric conditions

• Migraine or headache

• Other specified nonapproved indications (e.g., restless leg syndrome)^†^

• Dose over 1,800 mg / day

**Nonapproved indications not otherwise specified (e.g., message was “off-label uses,” “non anti-convulsant use,” or “growing uses”)**

**Other message content**

• Other comments that do not reference a specific indication or dose (e.g., side effects, pharmacokinetics, general comments on dosing)

*At the time of the visits, the only FDA-approved indication for gabapentin was for the adjunctive treatment of partial seizures in patients over age 12 y in doses ≤ 1,800 mg/day

^†^Other specified off-label uses included reflex sympathetic dystrophy, restless leg syndrome, essential tremor, and “neuritis”

While new forms of marketing such as direct-to-consumer advertising have captured the attention of the medical community, drug detailing remains a major form of pharmaceutical promotion, with expenditures of US$6.7 billion per year helping to pay tens of thousands of sales representatives (estimated at one representative for every 4.7 office-based physicians) [63,64]. We found that detail visits for gabapentin often involved messages about unapproved uses and were perceived as having high informational value. Despite the brevity of these visits, half of the respondents indicated their intention to increase their future prescribing or recommendation for the drug. In light of the potential for transmission of unbalanced information and the impact of detail visits on prescribing, the most prudent course of action may be for physicians to curtail or abstain from detail visits, and instead turn to the variety of free and low-cost resources that provide a more objective perspective on the merits and pitfalls of new drugs (http://www.ohsu.edu/drugeffectiveness) [65].
